# Bioinformatics research at BGRS-2018

**DOI:** 10.1186/s12859-018-2566-7

**Published:** 2019-02-05

**Authors:** Tatiana V. Tatarinova, Ming Chen, Yuriy L. Orlov

**Affiliations:** 1 Department of Biology, University of La Verne, La Verne, CA USA; 2 Department of Fundamental Biology and Biotechnology, Siberian Federal University, 660074 Krasnoyarsk, Russia; 3 Vavilov Institute of General Genetics RAS, Moscow, Russia; 4 A.A. Kharkevich Institute for Information Transmission Problems, Russian Academy of Sciences, Moscow, Russia; 5 Zhejiang University, Hangzhou, 310058 China; 6 Institute of Cytology and Genetics SB RAS, 630090 Novosibirsk, Russia; 7 Novosibirsk State University, 630090 Novosibirsk, Russia; 8 A.O. Kovalevsky Institute of Marine Biological Research of RAS, 119334 Moscow, Russia

This thematic issue of BMC Bioinformatics continues the series of BioMed Central special post-conference journal issues presenting materials from the bioinformatics and systems biology summits BGRS\SB (Bioinformatics of Genome Regulation and Structure\Systems Biology). BGRS/SB conference is biannually conducted in Novosibirsk since 1998. In this issue we present five selected papers from the XI^th^ BGRS\SB-2018 multi-conference (http://conf.bionet.nsc.ru/bgrssb2018/en/). This Special Issue is accompanied by other BioMed Central Special Issues collecting presented works in the fields of genomics, evolutionary biology, plant biology, and genetics, published as BMC Genomics, BMC Evolutionary Biology, BMC Systems Biology and BMC Plant Biology supplements [1–4]. Recent works at Belyaev Conference-2017 (“Belyaev Readings-2017” - http://conf.bionet.nsc.ru/belyaev100/en) in Novosibirsk, Russia were presented at BioMed Central as well [5–7]. BGRS\SB-2018 included several symposia: “Cognitive Sciences, Genomics and Bioinformatics” (SCGB-2018), “Systems biology and biomedicine” (SbioMed-2018), “Biodiversity: genomics and evolution” (BioGenEvo-2018), “Mathematical modeling and high-performance computing in bioinformatics, biomedicine and biotechnology” (MM-HPC-BBB-2018), “Systems biology of DNA repair processes and programmed cell death” (SbPCD-2018). Each session had presentations of bioinformatics applications.

Current special post-conference issue contains selected works on bioinformatics ranging from software for scientific literate mining to applications in plant biology. Below is a brief summary of the papers in this special issue.

Ivanisenko et al. [8] presented a new version of the popular ANDSystem tool for automatic text mining of scientific publications, this time equipped with expanded functionality. Currently, there is a number of commercial automated services allowing users to reconstruct molecular-genetic networks using the data automatically extracted from the texts of scientific publications, for example: STRING (https://string-db.org), Pathway Commons (https://www.pathwaycommons.org/), MetaCore (https://portal.genego.com/), and Ingenuity (https://www.qiagenbioinformatics.com/). Presented tool ANDSystem reconstructs associative gene networks taking into account the tissue-specific gene expression. The system allows the reconstruction of combined gene networks, as well as performing the filtering of genes tissue-specific expression. As an example of the application of such filtering, gene network of the extrinsic apoptotic signaling pathway was analyzed. Note that previous publication of this tool was at BMC Systems Biology [9], and recent applications was published in BMC Medical Genomics by Saik et al. [10].

Ranajit Das and Priyanka Upadhyai [11] showed application of the Geographic Population Structure (GPS) and reAdmix algorithms [12, 13] to biogeographic analyses of captive gorillas. The Geographic Population Structure algorithm is an admixture based tool for inference of provenance and has been previously employed for the geo-localization of various human populations worldwide [14–17]. Given the strong correspondence between geography and genetics, a number of strategies have focused on the delineation of the precise geographic origin of human populations using high-resolution genetic data. Das and Upadhyai [11] applied the GPS tool for localization of the ancestral origins of wild and captive gorilla genomes, of unknown geographic source, available in the Great Ape Genome Project [18]. Determination of the source population of captive gorillas can provide valuable information to guide breeding programs and ensure their appropriate management at the population level. Finally, the authors’ findings shine light on the broader applicability of GPS for protecting the genetic integrity of endangered non-human species.

Fedor Kazantsev and co-authors [19] presented an application in plant biology - the database on Molecular Identification of Genes for Resistance in Wheat (abbreviated as MIGREW). Wheat is one of the leading crops worldwide. The wheat pathogen complex affecting the plant organs containing chlorophyll is represented by the following species: *Puccinia triticina* causing leaf rust, *Puccinia graminis* causing stem rust, and *Blumeria graminis* causing powdery mildew. Population structure of fungal pathogens depends on environment and wheat ecotype. Taking into account the evolution of host-pathogen interactions, genetic diversity of wheat and fungus must be monitored. MIGREW database is developed using classical Model-View-Controller architecture. The model layer is the PostgreSQL database containing sixteen tables. The Controller layer is the Java application designed using *spring.io* libraries that performs REST API access to the data. The MIGREW database has been developed to present in single web-based interface the information on fungi-wheat objects keeping the data available for users with different requests, breeders and plant pathologists.

Kuzmin et al. [20] presented a result of a challenging project – assembly of the Siberian larch nuclear genome. Conifers have large genomes (~ 12–30 Gbp, which is 4–10 times larger than the human genome) containing ~ 80% of repetitive DNA. Using a new stepwise de novo assembling method presented in the paper, the genome of Siberian larch, *Larix sibirica* Ledeb. was for the first time completely assembled using de novo assembler by the CLC Assembly Cell. Sequencing and computational difficulties make it the first larch genome, and the sixths conifer genome assembly. The approach presented by Kuzmin et al. paves a road for assembling of very large genomes with a reasonable computing time and without engaging huge computing resources. The assemblies produced by this approach are of reasonable quality allowing their annotation and further use.

Evgenia Bondar and co-authors [21] analyzed chloroplast genome of the Siberian larch. Illumina sequencing reads were processed using the Bowtie2 [22] mapping program and assembled with the SPAdes genomic assembler [23]. Genome annotation was performed using the RAST server [24–26]. GMATo program was used for the SSRs search, and the Bowtie2 and UGENE [27] programs for the SNPs detection. This is the first effort to sequence and assemble the complete chloroplast genome sequence of Siberian larch. This assembly provides a reference for chloroplast resequencing and search for additional genetic markers using population samples. It will be useful for further phylogenetic and gene flow studies in conifers.

Therefore, this issue includes reports of recent bioinformatics application in text mining, animal genetics, database and algorithm development for computational plant sciences. BGRS\SB-2018 multi-conference had several parallel symposia, sessions and workshops, including First Sino-Russian Workshop on Integrative Bioinformatics and Systems Biology (http://conf.bionet.nsc.ru/srw2018/en/) and international Round table on education in bioinformatics. Other related computational biology works are presented in parallel BioMed Central issues by 2018. The conference was completed by Young Scientists School “Systems Biology and Bioinformatics” (SBB-2018) (http://conf.bionet.nsc.ru/bgrssb2018/en/school/). BioMed Central previously had published special issues by materials of SBB Schools. We invite our readers worldwide to attend our next event - Systems Biology and Bioinformatics Young Scientists School in summer 2019 in Novosibirsk, Russia.
